# Infant Outcomes Following Maternal Infection With Severe Acute Respiratory Syndrome Coronavirus 2 (SARS-CoV-2): First Report From the Pregnancy Coronavirus Outcomes Registry (PRIORITY) Study

**DOI:** 10.1093/cid/ciaa1411

**Published:** 2020-09-18

**Authors:** Valerie J Flaherman, Yalda Afshar, W John Boscardin, Roberta L Keller, Anne H Mardy, Mary K Prahl, Carolyn T Phillips, Ifeyinwa V Asiodu, Vincenzo Berghella, Brittany D Chambers, Joia Crear-Perry, Denise J Jamieson, Vanessa L Jacoby, Stephanie L Gaw

**Affiliations:** 1 University of California San Francisco, San Francisco, California, USA; 2 University of California Los Angeles, Los Angeles, California, USA; 3 Thomas Jefferson University, Philadelphia, Pennsylvania, USA; 4 National Birth Equity Collaborative; 5 Emory University, Atlanta, Georgia, USA

**Keywords:** SARS-CoV-2, COVID-19, pregnancy, newborn

## Abstract

Infant outcomes after maternal severe acute respiratory syndrome coronavirus 2 (SARS-CoV-2) infection are not well described. In a prospective US registry of 263 infants, maternal SARS-CoV-2 status was not associated with birth weight, difficulty breathing, apnea, or upper or lower respiratory infection through 8 weeks of age.

Maternal viral infection in pregnancy and the peripartum and postpartum periods can adversely affect infant outcomes. While studies have reported that maternal severe acute respiratory syndrome coronavirus 2 (SARS-CoV-2) infection increases the risk of preterm birth [1] and can be vertically transmitted [2–5], overall risks for infants born to mothers with SARS-CoV-2 are not yet well described. Currently, national and international guidelines for management of infants born to mothers with SARS-CoV-2 [6–8] are based on limited data without outcomes reported past the neonatal period. A more complete understanding of infant outcomes after maternal SARS-CoV-2 infection would inform guidelines and policies to manage this important and growing segment of the population.

To address this urgent need, we report here early findings from infants born to mothers enrolled in the Pregnancy Coronavirus Outcomes Registry (PRIORITY), an ongoing, nationwide study of pregnant or recently pregnant women who have confirmed or suspected SARS-CoV-2.

## METHODS

PRIORITY is a prospective cohort study enrolling US individuals ≥ 13 years old with suspected or confirmed SARS-CoV-2 during pregnancy or in the first 6 weeks after pregnancy. This manuscript reports infant outcomes for live births occurring to 179 mothers who had a positive test for SARS-CoV-2 and 84 mothers who had a negative test for SARS-CoV-2, and excludes live births of 10 mothers suspected of SARS-CoV-2 who were not tested. Maternal outcomes from PRIORITY will be reported separately.

Mothers were recruited nationally through outreach by professional organizations, traditional media, social media, and word of mouth to health-care providers. Once recruited, informed consent was obtained by the study team from the mother, for herself and her infant; births occurred at over 100 hospitals across the United States. PRIORITY was approved by the University of California San Francisco Institutional Review Board (IRB; #20–30410).

Clinical and demographic data were collected from mothers by phone, email, or text at the time of enrollment, after birth, and at 6–8 weeks after delivery, with participants reporting results of SARS-CoV-2 tests performed by their own providers. Consistent with the registry design, most outcomes were obtained by maternal report. For outcomes for which confirmation was crucial, including birth defects and positive infant testing for SARS-CoV-2, infant medical records were obtained from the electronic medical record of the hospital of birth to confirm the maternal report. In addition, convenience samples of 61 of 179 (34.1%) maternal reports of maternal SARS-CoV-2 and 24 of 44 (54.5%) maternal reports of neonatal intensive care unit (NICU) admission were also adjudicated from the medical record; all (100%) of these medical records correlated with the maternal report.

PRIORITY initiated enrollment on 22 March 2020, with a sample size of 1200 women justified by feasibility and an initial focus on gathering urgently needed data on birth defects, NICU admission, abnormal newborn examinations, positive infant tests for SARS-CoV-2, and respiratory complications at birth. Additional infant outcome items were IRB approved and added to both the birth questionnaire and the 6–8-week questionnaire on 13 May 2020. See Supplementary Table 1 for infant questionnaire items and their initial collection dates. PRIORITY enrollment and follow-up are ongoing; for this manuscript, we report data available by 22 June 2020.

We calculated the incidence and associated 95% confidence intervals for adverse outcomes using exact binomial techniques. We used a chi-square analysis and Fisher’s exact test to compare the proportions of outcomes between infants whose mothers tested positive for the virus and those whose mothers tested negative.

## RESULTS

Our cohort of 263 infants included 179 born to mothers testing positive for SARS CoV-2 and 84 born to mothers testing negative. Among those testing positive, 146 (81.6%) were symptomatic, while among those testing negative, 53 (63.1%) were symptomatic (*P* = .001). See Table 1 for other clinical and demographic characteristics by maternal SARS CoV-2 status.

**Table 1.** Maternal and Infant Characteristics, by Maternal Severe Acute Respiratory Syndrome Coronavirus 2 Status

	SARS-CoV-2 positive, n (%) or mean (SD), n = 179	SARS-CoV-2 negative, n (%) or mean (SD), n = 84	*P* value
Maternal age^a^	31.5 ± 5.4	31.7 ± 5.2	.78
Maternal race/ethnicity^a^			
American Indian/Alaskan Native	0 (0)	0 (0)	
Asian	14 (7.9)	6 (7.3)	.87
Black or African American	20 (11.3)	6 (7.3)	.32
Hispanic or Latina	49 (27.7)	17 (20.7)	.23
Native Hawaiian or other Pacific Islander	1 (.6)	1 (1.2)	.58
White	103 (58.2)	59 (72.0)	.033
Mother college graduate^a^	99 (56.1)	57 (69.5)	.33
Income ≥$100 000^a^	71 (40.1)	38 (46.3)	.87
Geographic region^a^	…	…	<.0001
Midwest	46 (26.1)	16 (19.5)	
Northeast	58 (33.0)	11 (13.4)	
South	32 (18.2)	11 (13.4)	
West	40 (22.7)	44 (53.7)	
Mother hospitalized at time of study enrollment^a^	18 (10.2%)	7 (8.5%)	.67
Mother in intensive care unit at time of study enrollment^a^	6 (3.4%)	0 (0%)	.09
Gestational age at birth^b^	38.3 ± 2.6	38.2 ± 2.8	.77
Preterm birth^b^	21 (13.9)	9 (16.1)	.69
Birthweight, grams^c^	3211 ± 738	3198 ± 831	.99
Delivered vaginally	106 (59.2)	50 (59.5)	.79
Primiparous mothers	79 (44.1)	32 (38.1)	.36
Female infant^d^	42 (52.5)	17 (39.5)	.17
Birth defect	2 (1.1)	1 (1.2)	.96
Breast milk after birth	148 (83.1)	77 (91.7)	.07
NICU admission	31 (17.3)	13 (15.5)	.71
Abnormal newborn exam	3 (1.7)	1 (1.2)	.76
Infant positive for SARS-CoV-2	2 (1)	0 (0)	.17
Fast or difficulty breathing^d^	11 (13.8)	3 (7.0)	.26
Upper respiratory infection^e^	2 (5.0)	1 (6.3)	.85
Apnea^e^	0 (0)	2 (4.7)	.052
Rooming in with mother^d^	57 (71.3)	34 (79.1)	.35
Breastfeeding at 6–8 weeks^e^	35 (87.5)	14 (87.5)	1.00

Abbreviations: NICU, neonatal intensive care unit; SARS-CoV-2, severe acute respiratory syndrome coronavirus 2; SD, standard deviation.

^a^This item was assessed among 176 and 82 infants of mothers testing positive or negative, respectively, for SARS-CoV-2.

^b^This item was assessed among 151 and 56 infants of mothers testing positive or negative, respectively, for SARS-CoV-2.

^c^This item was assessed among 173 and 83 infants of mothers testing positive or negative, respectively, for SARS-CoV-2.

^d^This item was assessed among 80 and 43 infants of mothers testing positive or negative, respectively, for SARS-CoV-2.

^e^This item was assessed among 40 and 16 infants of mothers testing positive or negative, respectively, for SARS-CoV-2.

In this cohort of 263 infants, 44 infants (17%) were admitted to the NICU; fast breathing or difficulty breathing was reported for 14 of 127 (11%) infants surveyed after expansion of the birth questionnaire, and apnea was reported for 2 (1.6%). These characteristics did not differ between mothers testing positive for SARS-CoV-2 compared to those who tested negative. Among infants born to mothers who first tested positive 0–14 days prior to delivery, 20 of 77 (26.0%) were admitted to the NICU, compared to 10 of 82 (12.2%) born to mothers who first tested positive more than 14 days prior to delivery (*P* = .04). Infants born to mothers who first tested positive 0–14 days prior to delivery were also born earlier, as compared to infants born to mothers who first tested positive more than 14 days prior to delivery (mean 37.5 versus 39 weeks of gestation, respectively; *P* = .0009). Additionally, in this cohort, 16 mothers first tested positive for SARS-CoV-2 after delivery; the positive tests for this subgroup occurred a median of 6 days after delivery, with an interquartile range of 1–12 days after delivery. Infants born to mothers who first tested positive 0–14 days prior to delivery were less likely to room in with mothers than were those born to mothers who first tested positive more than 14 days prior to delivery or after delivery (see Supplementary Table 2).

Of the infants born to mothers who tested positive for SARS-CoV-2 in the third trimester, 2 were reported to have birth defects, each with multiple congenital anomalies reported: 1 infant had cardiac, vertebral, renal, and pulmonary anomalies, while the other had facial, genital, renal, brain, and cardiac anomalies. Infant gastrointestinal, renal, and cardiac anomalies were also reported by 1 mother who tested negative for SARS-CoV-2.

Through 6–8 weeks of age, no pneumonia or lower respiratory tract infection was reported. Among 56 infants assessed at 6–8 weeks of age for an upper respiratory infection (URI), a URI was reported for 2 (5.0%) infants of SARS-CoV-2–positive mothers and 1 (6.3%) infant of a SARS-CoV-2–negative mother (*P* = .85). In the first 6–8 weeks of follow-up, 2 (1.1%) infants born to SARS-CoV-2–positive mothers tested positive for SARS-CoV-2, and 1 had an indeterminate SARS-CoV-2 test. Of these, 1 was a late preterm infant with a positive nasopharyngeal swab for SARS-CoV-2 at 24 hours of life and a negative nasopharyngeal swab for SARS-CoV-2 at 48 hours of life; this infant breastfed and roomed in after birth, and was discharged home at 2 days of life. The other was born at 26 weeks gestation and received expressed donor milk. This infant’s initial SARS-CoV-2 test was negative at 24 hours of life, positive at 48 hours of life, and negative at 6 and 8 days of life; she had a clinical course notable for mild lymphocytosis, anemia, and unilateral corneal opacification of unknown etiology, and was otherwise typical of her gestational age. The estimated incidence of a positive test for SARS-CoV-2 among infants of mothers with positive tests for SARS-CoV-2 was 1.1% (0.1%, 4.0%). No infant required rehospitalization in the follow-up period.

## Discussion

Among 263 initial infants enrolled in the PRIORITY study, adverse outcomes, including preterm birth, NICU admission, and respiratory disease, did not differ between those born to mothers testing positive for SARS-CoV-2 and those born to mothers testing negative. No pneumonia or lower respiratory tract infection was reported in this cohort through 6–8 weeks of age. Among infants born to mothers who tested positive for SARS-CoV-2, the estimated incidence of a positive infant SARS-CoV-2 test was low, at 1.1% (0.1%, 4.0%), and infants had minimal symptoms. Overall, these results are reassuring and suggest that infants born to mothers infected with SARS-CoV-2 generally do well in the first 6–8 weeks after birth.

Our study has several limitations. First, we are unable to estimate the incidence of infant SARS-CoV-2 infection, because infant testing was incomplete and might be biased by both false-positive and false-negative results. Further research is needed to report the infant incidence of SARS-CoV-2 after maternal infection. Second, since PRIORITY’s control group includes both symptomatic and asymptomatic women testing negative for SARS-CoV-2, it may not be representative of all US pregnancies. However, these inclusion criteria allowed sampling of control mothers who were more similar to the exposed group in all respects except for SARS-CoV-2 test results, which may enhance causal inference for the effect of SARS-CoV-2 on infant outcomes. Third, PRIORITY’s current racial and ethnic distribution underrepresents maternal Latina ethnicity and Black race compared to a concurrent Centers for Disease Control and Prevention assessment of US pregnant women infected with SARS-CoV-2, which reported race/ethnicity as 46% Hispanic, 22% Black, and 23% White [9]. Barriers to registry participation are expected given the historical harm related to research participation and systemic racism experienced by Black, indigenous, and people of color communities and the current burden of SARS-CoV-2 in these communities, and may impact the generalizability of our findings [10]. In May 2020, PRIORITY launched a Reproductive Health Equity and Birth Justice Core to increase enrollment of underrepresented groups and engage with partners in highly impacted communities. Fourth, the timing of maternal testing in this cohort was determined at the clinical sites and may not have coincided with the onset of illness. Therefore, while we found that NICU admission and earlier gestational age were more common for infants born to mothers testing positive for SARS-CoV-2 at 0–14 days before delivery than for those testing positive at other times, these associations may reflect hospital practices for management of mothers testing positive for SARS-CoV-2 rather than infant physiology.

Overall, PRIORITY’s initial findings regarding infant health are reassuring. Further investigation with longer follow-up periods and larger sample sizes will be needed to make a definitive determination of the risks of vertical transmission and neonatal illness and the incidences of congenital anomalies, and are planned for the PRIORITY cohort.

## Supplementary Data

Supplementary materials are available at *Clinical Infectious Diseases* online. Consisting of data provided by the authors to benefit the reader, the posted materials are not copyedited and are the sole responsibility of the authors, so questions or comments should be addressed to the corresponding author.

ciaa1411_suppl_Supplemental-Tables

## Notes

***Acknowledgments.*** The authors thank all of the study participants.

***Financial support.*** This work was supported by the University of California San Francisco National Center of Excellence in Women’s Health; California Health Care Foundation; the Centers for Disease Control and Prevention Foundation; the Bill and Melinda Gates Foundation; and individuals that provided support through 2 crowdfunding sites (https://givingtogether.ucsf.edu/fundraiser/2718761 and https://spark.ucla.edu/project/20775).

***Potential conflicts of interest*.** S. L. G. received grant K08AI141728 from the National Institutes of Health, outside the submitted work. V. J. F. reports grants from the Bill and Melinda Gates Foundation and a contract with California Department of Health Care Services, outside the submitted work. B. D. C. reports grants from Tara Health Foundation and Robert Wood Foundation, outside the submitted work. All other authors report no potential conflicts.All authors have submitted the ICMJE Form for Disclosure of Potential Conflicts of Interest. Conflicts that the editors consider relevant to the content of the manuscript have been disclosed.
