# Supplementary material for: Infant Outcomes Following Maternal Infection With Severe Acute Respiratory Syndrome Coronavirus 2 (SARS-CoV-2): First Report From the Pregnancy Coronavirus Outcomes Registry (PRIORITY) Study
Source: Clin Infect Dis. 2020 Sep 18;73(9):e2810–3. doi: 10.1093/cid/ciaa1411 (PMC7543372; doi:10.1093/cid/ciaa1411)
Supplement: ciaa1411_suppl_Supplemental-Tables [file ciaa1411_suppl_Supplemental-Tables.docx]

**Supplemental Table 1: Items used to assess infant outcomes in the PRIORITY study and the date of their deployment**

|  | Date of Implementation | Time of Administration |
| --- | --- | --- |
| What date did your pregnancy end? | 3/21/2020 | At First Report of End of Pregnancy |
| What date is your baby due? | 3/21/2020 | At Maternal Enrollment |
| What did your baby weigh at birth? | 3/21/2020 | At First Report of End of Pregnancy |
| Did you deliver vaginally, by Cesarean or both? | 3/21/2020 | At First Report of End of Pregnancy |
| How many previous live births have you had? | 3/21/2020 | At Maternal Enrollment |
| What is the infant's sex? | 5/20/2020 | At First Report of End of Pregnancy |
| Has your infant breastfed or received any breast milk? | 3/21/2020 | At First Report of End of Pregnancy |
| Did your baby have any of the following problems…? |  | At First Report of End of Pregnancy |
| - Abnormal findings on the newborn exam | 3/21/2020 | At First Report of End of Pregnancy |
| - Admission to neonatal intensive care unit? | 3/21/2020 | At First Report of End of Pregnancy |
| - Apnea | 5/20/2020 | At First Report of End of Pregnancy |
| - Baby diagnosed with COVID-19 | 3/21/2020 | At First Report of End of Pregnancy and At 6/8 week Follow-up |
| - Birth defect (specify) | 3/21/2020 | At First Report of End of Pregnancy |
| - Breathing support after birth (need help breathing with mask or breathing tube) | 3/21/2020 | At First Report of End of Pregnancy |
| - Fast breathing or difficulty breathing | 5/20/2020 | At First Report of End of Pregnancy |
| Since being discharged from the birth hospitalization, has your baby had a stuffy nose, runny nose, increased sneezing or cough without a change in her/his breathing (a cold, or upper respiratory tract infection)? | 5/20/2020 | At 6/8 week Follow-up |
| Since being discharged from the birth hospitalization, has your baby had wheezing or a change in her/his breathing with or without cough or fever (lower respiratory tract infection, bronchiolitis, wheezing illness, pneumonia)? | 5/20/2020 | At 6/8 week Follow-up |
| Did you and your infant "room in" (share the same hospital room) while in the hospital? | 5/20/2020 | At First Report of End of Pregnancy |
| Are you currently breastfeeding or providing breast milk to your baby? | 5/20/2020 | At 6/8 week Follow-up |

**Supplemental Table 2: Outcomes among infants of mothers testing positive for SARS-CoV-2, by timing of diagnosis**

| \|  \| Mothers first testing positive for SARS-CoV-2 more than 14 days before delivery (n=82) \| Mothers first testing positive for SARS-CoV-2 0-14 days before delivery (n=77) \| Mothers first testing positive for SARS-CoV-2 after delivery (n=16) \| p-value \| \| --- \| --- \| --- \| --- \| --- \| \| Gestational age (wks) (mean, SD)^a^ \| 39.0$\pm$2.2 (n=77) \| 37.5$\pm$2.9 (n=57) \| 37.8$\pm$2.7 (n=13) \| 0.0002^1^ \| \| Gestational age (n, %)^a^ \|  \|  \|  \| 0.087 \| \| >=39 wks \| 55 (71.4) \| 25 (43.9) \| 6 (46.2) \|  \| \| 37-38 6/7 wks \| 15 (19.5) \| 20 (35.1) \| 5 (38.5) \|  \| \| 34-36 6/7 wks \| 4 (5.2) \| 6 (10.5) \| 1 (7.7) \|  \| \| 28-33 6/7 wks \| 2 (2.6) \| 6 (10.5) \| 1 (7.7) \|  \| \| <28 wks \| 1 (1.3) \| 0 (0) \| 0 (0) \|  \| \| Birth weight (g) (mean, SD)^b^ \| 3301$\pm$ 643 \| 30$68\pm$832 \| 33$51\pm$ 634 \| 0.051 \| \| Admitted to Neonatal Intensive Care Unit (n, %) \| 10 (12.2) \| 20 (26.0) \| 1 (6.3) \| 0.034 \| \| Roomed in with mother (n, %)^c^ \| 45 (81.8) \| 9 (42.9) \| 2 (66.7) \| 0.004 \| |
| --- | --- | --- | --- | --- | --- | --- | --- | --- | --- | --- | --- | --- | --- | --- | --- | --- | --- | --- | --- | --- | --- | --- | --- | --- | --- | --- | --- | --- | --- | --- | --- | --- | --- | --- | --- | --- | --- | --- | --- | --- | --- | --- | --- | --- | --- | --- | --- | --- | --- | --- | --- | --- | --- | --- | --- |

^a^This item assessed among 77, 57 and 13 infants of mothers testing positive for SARS-CoV-2 more than 14 days before delivery, 0-14 days before delivery, and after delivery, respectively; ^b^This item assessed among 81, 72 and 16 infants of mothers testing positive for SARS-CoV-2 more than 14 days before delivery, 0-14 days before delivery, and after delivery, respectively; ^c^This item assessed among 55, 21 and 3 infants of mothers testing positive for SARS-CoV-2 more than 14 days before delivery, 0-14 days before delivery, and after delivery, respectively
